# Haematopoietic Stem Cell Transplantation in Adolescents and Young Adults With Acute Lymphoblastic Leukaemia: Special Considerations and Challenges

**DOI:** 10.3389/fped.2021.796426

**Published:** 2022-01-11

**Authors:** Charlotte Calvo, Leila Ronceray, Nathalie Dhédin, Jochen Buechner, Anja Troeger, Jean-Hugues Dalle

**Affiliations:** ^1^Pediatric Hematology and Immunology Department, Robert Debré Academic Hospital, GHU APHP Nord - Université de Paris, Paris, France; ^2^Department of Pediatric Hematology and Oncology, St. Anna Children's Hospital, Medical University of Vienna, Vienna, Austria; ^3^Hematology for Adolescents and Young Adults, Saint-Louis Academic Hospital GHU APHP Nord - Université de Paris, Paris, France; ^4^Department of Pediatric Hematology and Oncology, Oslo University Hospital, Oslo, Norway; ^5^Department of Pediatric Hematology, Oncology and Stem Cell Transplantation, University Hospital of Regensburg, Regensburg, Germany

**Keywords:** acute lymphoblastic leukaemia, adolescent, young adult, haematopoietic stem cell transplant, conditioning regimen, supportive care, transition to adult care

## Abstract

Adolescents and young adults (AYAs) represent a challenging group of acute lymphoblastic leukaemia (ALL) patients with specific needs. While there is growing evidence from comparative studies that this age group profits from intensified paediatric-based chemotherapy, the impact and optimal implementation of haematopoietic stem cell transplantation (HSCT) in the overall treatment strategy is less clear. Over recent years, improved survival rates after myeloablative allogeneic HSCT for ALL have been reported similarly for AYAs and children despite differences in transplantation practise. Still, AYAs appear to have inferior outcomes and an increased risk of treatment-related morbidity and mortality in comparison with children. To further improve HSCT outcomes and reduce toxicities in AYAs, accurate stratification and evaluation of additional or alternative targeted treatment options are crucial, based on specific molecular and immunological characterisation of ALL and minimal residual disease (MRD) assessment during therapy. Age-specific factors such as increased acute toxicities and poorer adherence to treatment as well as late sequelae might influence treatment decisions. In addition, educational, social, work, emotional, and sexual aspects during this very crucial period of life need to be considered. In this review, we summarise the key findings of recent studies on treatment approach and outcomes in this vulnerable patient group after HSCT, turning our attention to the different approaches applied in paediatric and adult centres. We focus on the specific needs of AYAs with ALL regarding social aspects and supportive care to handle complications as well as fertility issues. Finally, we comment on potential areas of future research and concisely debate the capacity of currently available immunotherapies to reduce toxicity and further improve survival in this challenging patient group.

## Introduction

Adolescents and young adults (AYAs) with acute lymphoblastic leukaemia (ALL) are a unique group of patients at the interface between childhood and adulthood (1). There is no consensus on the definition of AYAs: the World Health Organisation defines adolescents as individuals of 10–19 years old, while the National Cancer Institute defines the AYA population as 15–39 years old (2, 3). In Europe, patients are considered an AYA if they are aged 15–29 years (2). These differences in age definition affect access to different types of care structure, clinical trials and treatment protocols (2).

Although the survival rate now approaches 90% for childhood ALL, the prognosis remains poorer in AYAs (2, 3). In fact, survival of ALL is triphasic during adulthood, with survival rates of 75% when treated at 17 years, 48% at 20 years, and 15% at 70 years—also known as the “survival cliff” (4, 5).

The inferior prognosis of ALL in AYAs compared to in children can be explained in part by the age-related variations in the molecular subtypes of ALL (Table 1) (6). The frequency of ALL with a T-cell phenotype is about twice higher in AYA compared to younger children (<15 years old) (7). The prevalence of hyperdiploidy and *ETV6-RUNX1*-positive ALL—both of which are associated with good prognosis—declines from 25 to 30% in children to <3% in young adults (aged 21–39 years) (4, 6, 7). Conversely, the prevalence of Philadelphia chromosome (Ph)-positive ALL—which is associated with poor prognosis—is markedly increased in patients aged 21–39 years vs. in children (4, 6, 7). Also, the prevalence of Ph-like ALL (poor prognosis) rises with age, from 10 to 15% in children with B-cell ALL to over 25% in young adults with ALL (5, 6, 8). Some genomic abnormalities have a peak incidence in the AYA population e.g., *iAMP21, DUX4* rearrangement, *ZNF384* rearrangement, and *MEF2D* rearrangement (4). Age-related variation of genomic abnormalities from childhood to AYA B-cell precursor ALL (BCP-ALL) are represented in Table 1.

**Table 1:** Genomic landscape of BCP-ALL: childhood vs. AYA.

	**Childhood**	**AYA**
ETV6-RUNX1	25%	<5%
Hyperdiploid	25%	<5%
TCF3-PBX1	5%	<5%
KMT2A rearranged	<5%	5–10%
DUX4-ERG	5%	15%
Hypodiploid	<1%	5%
Ph positive	2–5%	5–10%
Ph like	10–15%	25–30%
ZNF384	5%	10%
MEF2D	5%	7%
iAMP21	1–5%	6–12%
Other	5–10%	10–15%

The survival cliff between the ages of 17 and 21 years has also been attributed to the transition of patients from paediatric to adult treatment sites and protocols (5). Superior survival has been consistently demonstrated in various countries when AYA patients are treated using paediatric chemotherapy protocols rather than adult ones, with differences in type and intensity of anti-leukaemic drugs vs. adult protocols (1, 2, 5, 7, 9, 10). In fact, more extensive use of glucocorticoids, vincristine and pegylated asparaginase with intensive and prolonged central nervous system (CNS) prophylaxis results in survival benefit for AYA patients with ALL (10, 11). Indeed, in the largest published cohort of patients 1–45 years of age treated with the same ALL frontline protocol (NOPHO ALL-2008), event-free survival (EFS) and overall survival (OS) for 18–45-year-old patients with Ph-negative ALL were 74 and 78%, respectively (12). For high-risk patients with or without an indication for haematopoietic stem cell transplantation (HSCT), no significant difference in 5-year EFS was seen between the three age groups: 1–9, 10–17, and 18–45 years (12). In keeping with these findings, Wieduliwilt et al. concluded from their study that post-remission therapy with paediatric-style chemotherapy was superior to myeloablative conditioning followed by allogeneic HSCT in AYA patients with Ph-negative ALL in first complete remission (CR1) in terms of OS (66 vs. 45%, respectively), disease-free survival (DFS; 58 vs. 44%, respectively), and non-relapse mortality (NRM; 8 vs. 29%, respectively) (13).

However, access to clinical trials and paediatric regimens is not readily available to all AYA patients (4).

The better OS and lower relapse rate associated with treatment of AYAs on paediatric wards may be due to higher therapy intensity and stricter adherence to chemotherapy schedules. Conversely, being treated on adult wards may lead to better and earlier access to novel therapies not yet available in paediatric centres in patients with refractory disease.

The use of paediatric-inspired or fully paediatric strategies has improved outcomes in AYAs with Ph-negative ALL and, as a consequence, has led experts to question allogeneic HSCT indications in this population (2). Because of its associated short- and long-term toxicities, progress in chemotherapy management, and the advent of immunotherapy, HSCT could be reserved for AYA patients with ALL exhibiting early resistance to chemotherapy assessed by predefined evaluations of minimal residual disease (MRD), as recommended in paediatric protocols (2, 12, 14–16). At least in CR1, in AYA as in other patient-age subgroups MRD represents one of the most important point for identifying patients requesting treatment intensification (2, 12, 14–16). In addition, with the use of combined tyrosine kinase inhibitors (TKIs) and chemotherapy treatment, a further reduction in the proportion of patients eligible for HSCT has been achieved, even in the high-risk group (17–19).

Many adult patients with ALL treated on an adult regimen receive HSCT during first complete remission (CR1) if a matched donor is available. However, the ability to avoid the toxicities, late adverse effects, and financial costs of HSCT substantially favours paediatric regimens (5).

Toxicities represent a major issue in AYA patients with ALL. The use of intensified regimens raises the need for monitoring and preventing acute and late side effects that can affect survival and quality of life (2). Toxicities also represent a significant problem in AYA patients who undergo HSCT, with studies reporting 10–30% treatment-related mortality (TRM), which is higher than reported for younger patients and is mostly due to graft-vs.-host disease (GvHD) and infection (3). Hypofertility/infertility is a particularly relevant late side effect in AYAs (2). Many studies have shown the higher risk of non-adherence during therapy or follow-up in the AYA population vs. their younger counterparts (2).

Therefore, AYA patients undergoing HSCT to treat ALL represent a unique group with medical, psychological, and social issues requiring diligent care and follow-up. We try to address these points in this review. We fully acknowledge that the published literature uses different age definitions for AYAs, which makes comparisons cumbersome; but at least when reporting our own experience, we consider AYA patients to be aged between 15 and 29 years.

## Indication for HSCT

Beyond discussions about the definition of the AYA age group, we were not able to identify any papers specifically dedicated to allogeneic HSCT in an AYA ALL population. Published ALL studies reported overall results of ALL therapy in this population but did not necessarily disclose how an AYA group was defined, state whether there were any adjustments in the HSCT indications for this group compared with indications for children or adults, or address HSCT results specifically in AYAs (15, 20). In paediatric studies, AYAs are often limited to 18–21-year-old patients and results are not provided for older AYAs (20). In adult studies, AYAs are usually included in the overall cohort along with 40–60 years old patients; this probably worsen their outcome vs. if the older patients were studied separately.

Despite continuous improvement, overall results of ALL treatment in AYAs (including the results of initial chemotherapy, not only HSCT) appear to be worse than those obtained in younger children with the same protocols (3, 15). Differences in OS may relate to a higher incidence of TRM, especially from infections and GvHD (3, 15). However, none of the published registry or centre-based analyses reported in detail potential differences in HSCT indications and donor choice between the paediatric and AYA populations, leading to possible bias in the interpretation of results. Many studies report results based on donor allocation, i.e., a matched sibling donor (MSD) or matched unrelated donor (MUD) matched at 10 out of 10 human-leukocyte antigen (HLA) loci, rather than based on the final treatment received (chemotherapy alone vs. chemotherapy followed by HSCT, regardless of donor type) (3, 11). For patients in CR1, most current paediatric and adult protocols reserve HSCT for AYAs with high-risk/very -high-risk ALL defined by disease characteristics such as ALL cell origin (B cell or T cell), initial leukocyte count, cytogenetics and molecular profile at diagnosis, and response after induction with or without consolidation courses evaluated by MRD measurement using real-time polymerase chain reaction (RQ-PCR) or flow cytometry (3, 11, 21). In some adult protocols, the transplant indication is based on only the persistence of positive MRD at defined time points regardless of other disease characteristics (21). For children or adults, definitions of high-risk cytogenetics and molecular profiles differ by protocol. Since the molecular profile of paediatric and AYA patients differs, with AYAs patients having a poorer-risk profile (see above), a higher percentage of AYAs are allocated to HSCT, logically (3, 11). In addition, the anticipated toxicities of chemotherapy are not identical for the AYA and paediatric population (3). Acute toxicity is more prevalent in AYAs as compared with in paediatric patients. Therefore, clinicians are often quick to propose HSCT for AYA patients in CR1 in order to avoid the risk of death related to second-line chemotherapy if relapse occurs. In contrast, long-term sequalae of HSCT in paediatric patients represent a major concern leading to the avoidance of HSCT in CR1 in as many patients as possible. Paediatric protocols propose HSCT in <5–8% of patients in CR1, while adult protocols enrolling AYA propose HSCT in about 30% of patients in CR1 (22). Adult protocols also propose that patients in second complete remission (CR2) undergo HSCT from any available donor as long as the patient is <40 years old.

In the future, the use of immunotherapy such as inotuzumab ozogamicin and blinatumomab as well as chimeric antigen receptor (CAR) T-cell therapy may change the current HSCT algorithm in the AYA population (23–25).

## Choice of Conditioning Regimen

In allogeneic HSCT for ALL, older age is associated with poorer survival (3, 15). In most studies, age does not impact risk of relapse but is associated with increased TRM and GvHD: incidence of toxic death is more frequent in adults compared with in AYAs, and in AYAs compared with in children (3, 15, 26, 27). Thus, in the study of the Centre for International Blood and Marrow Transplant Research (CIBMTR) including patients transplanted for ALL between 2002 and 2007, 5-year TRM was 19% in children, 31% in AYAs, and 41% in older adults (15).

Reduced-intensity conditioning (RIC) regimens offer the advantage of decreased TRM but are often associated with a higher relapse rate (28). This option is being evaluated for patients over 45 years old in the Group for Research on Adult Acute Lymphoblastic Leukaemia (GRAALL) 2014 protocol. To date, there are no prospective studies comparing outcomes of patients with ALL who undergo myeloablative conditioning vs. RIC, but several large retrospective cohorts report the above-mentioned observations.

In a CIBMTR study, Marks et al. examined the role of the RIC regimens in adults over 35 years old transplanted in 1995–2006 for Ph-negative ALL. The age-adjusted OS, TRM and relapse rates were not statistically different between patients undergoing myeloablative conditioning vs. those undergoing RIC (29). In a European Group for Blood and Marrow Transplantation (EBMT) study, including patients over 45 years, RIC was associated with lower TRM and higher relapse rate than was myeloablative conditioning but this did not translate into a significant difference in OS (28). However, results of these retrospective studies should be interpreted with caution due to the heterogeneity of populations in terms of age at transplant, comorbidities, status at transplant, donor source, GvHD strategies, and RIC regimens used. In summary, although RIC could be a suitable alternative to myeloablative conditioning for older adults with ALL, there are no strong data to support a recommendation of this approach in the AYA population.

Total body irradiation (TBI) is widely used in myeloablative conditioning regimens for patients with ALL. Because TBI is associated with early and late adverse effects, transplant with TBI-free conditioning regimens has been evaluated in ALL patients. A small, randomised controlled trial found significantly higher EFS with TBI, etoposide and cyclophosphamide vs. busulfan, etoposide, and cyclophosphamide conditioning in paediatric ALL patients (30). Superiority of TBI over busulfan-based conditioning regimens has been also reported in retrospective studies both in children and in adults (31–34). Recently, the randomised, international, multicentre, Phase III For Omitting Radiation Under Majority age (FORUM) study investigated whether preparative combination chemotherapy could replace TBI in paediatric patients with ALL (35). The study randomised 417 patients aged 4–21 years at transplantation and in complete remission of ALL to myeloablative conditioning with either fractionated 12 Gy TBI and etoposide or with fludarabine, thiotepa and either busulfan or treosulfan. In the intention-to-treat population, 2-year OS was significantly higher following TBI (0.91; 95% confidence interval [CI], 0.86–0.95; *p* < 0.001) vs. chemo-conditioning (0.75; 95% CI, 0.67–0.81). A major difference was seen in the relapse rate, which was strongly decreased using TBI with a 2-year cumulative incidence of relapse of 0.12 (95% CI, 0.08–0.17) vs. 0.33 (95% CI, 0.25–0.40) following chemo-conditioning (*p* < 0.001). TRM was low in both arms, with a significant advantage for the TBI group: 2-year cumulative incidence of TRM was 0.02 (95% CI, 0.01–0.05) after TBI vs. 0.09 (95% CI, 0.05–0.14) after chemo-conditioning (*P* = 0.02). The superiority of TBI over chemotherapy regarding OS was observed both in patients aged 6–10 years and in patients aged 11–21 years (35).

Although the advantage of TBI has not been investigated or demonstrated specifically in an AYA population aged 15–29 years of age, the data above are in favour of TBI-based myeloablative conditioning for AYAs.

## Donor Source

### Peripheral Blood Stem Cells vs. Bone Marrow

In adults, the source of haematopoietic stem cells is mostly the peripheral blood (PBSC) in both MRD and MUD transplantation (36). Several prospective randomised studies comparing PBSC and bone marrow transplants following myeloablative conditioning have shown that PBSCs were associated with a decreased relapse rate in haematological malignancies and improved OS and DFS in patients with advanced-stage disease but not in those with early-stage disease. However, PBSC transplantation was also associated with a significant risk of extensive chronic GvHD (cGvHD) (37, 38).

In contrast, bone marrow remains the most common source used as an allograft in children with hematologic malignancies. Data regarding the association between HSCT outcome and stem-cell source in paediatric patients are limited and the role of PBSCs is debated. In a retrospective study on behalf of the EBMT Paediatric Diseases Working Party comparing HSCT outcomes either after bone marrow or PBSC allograft in children and adolescents <18 years transplanted for ALL in CR1 or CR2, the OS was significantly poorer after PBSC allograft compared with after bone marrow allograft due to a higher incidence of cGvHD and higher risk of NRM without improvement of relapse risk (38).

To date, it has been difficult to definitively conclude which is the best stem-cell source in AYAs transplanted for ALL but the studies cited above lead to a preference for bone marrow in most transplantations for early-stage disease (i.e., mainly in CR1).

### Alternative Donors: Unrelated Cord Blood or Haploidentical Donor

Unmanipulated haploidentical HSCT, other haploidentical HSCT technics and unrelated cord blood (UCB) transplantation may be alternative options to treat patients with high-risk ALL who do not have an HLA-matched donor (39, 40). To date, UCB transplantation has been used mostly in children, while T-cell repleted haploidentical transplant with high-dose post-transplant cyclophosphamide (PTCy) for GvHD prophylaxis has been widely used in adults. The EBMT conducted a retrospective study comparing outcomes in ALL adults patients after transplantation with UCB (*n* = 370) or an unmanipulated haploidentical graft with PTCy (*n* = 158) (41). In the multivariate analysis, UCB transplantation was associated with a lower incidence of cGvHD (hazard ratio [HR], 0.58; *P* = 0.01 for ALL) vs. an unmanipulated haploidentical graft. No difference was observed for relapse incidence (HR, 0.82; *P* = 0.31 for ALL), NRM (HR, 1.23; *P* = 0.23 for ALL) and leukaemia-free survival (HR, 1.00; *P* = 0.84 for ALL) between groups (41). In 2016, Michel et al. reported a randomised prospective study comparing the results of HSCT from either one or two UCB units in 137 paediatric and AYA patients (<35 years) with either ALL or acute myeloid leukaemia (39). Two-year post-transplant survival, DFS and TRM were 68.8% (CI 95%, ± 6.0%), 67.6% (CI 95%, ± 6.0%), and 5.9% (CI 95%, ± 2.9%), respectively, after single-unit transplantation compared with 74.8% (CI 95%, ± 5.5%, 68.1% (CI 95%, ± 6.0%), and 11.6% (CI 95%, ± 3.9%), respectively after double-unit transplantation (*P* = not significant).

Studies comparing manipulated haploidentical stem cell grafts (T-cell depleted) with unmanipulated haploidentical grafts and/or UCB grafts have not been done in adults or children. To date, no data are available on which to base a recommendation regarding which one of these two donor types (haplo-identical donor or UCB) is preferable in AYAs with ALL.

Table 2 summarises these results.

**Table 2:** Summary of published studies including AYA about HSCT in ALL.

**References**	**Definition of AYA**	**number of AYA/number of patients**	**Aim of the study**	**Type of conditioning: MAC TBI-based, MAC w/o TBI, RIC**	**Donor type: MSD, MUD, MMUD, haplo**	**Donor source: BM, PBSC, UCB**	**Principal outcomes**
Balduzzi et al. (42)	12–18 y/o	116/348	Transplantation in Children and Adolescents with Acute Lymphoblastic Leukaemia from a Matched Donor vs. an HLA-Identical Sibling	• 88% of MSD graft recipients and 86% of MUD graft recipients: MAC TBI-based. • The others: MAC w/o TBI	• MUD (43) • MSD (44)	• MSD: BM in 83%, PB in 15%, and CB in 2% • MUD: BM in 51%, PB in 43%, CB in 6%	Risk of c-GVHd was double in patients age >12 years than in patients age <12 years (HR, 2.35; 95% CI, 1.36–4.08; *P =* 0.002) Risk of death was higher in patients age >12 years compared with those age 2–12 years (HR, 1.62; 95% CI, 1.04–2.53; *P* = 0.034) Risk of non- leukemic death was almost 3-fold higher in patients age >12 years compared with those age <12 years (HR, 2.91; 95% CI, 1.50–5.65; *P* = 0.001) Among patients age >12 years: • 4-year EFS was 59 ± 8% for MSD graft recipients and 62 ± 6% for MUD graft recipients (*P =* 0.806), • 4-year NRM was 19 ± 7% for the former and 20 ± 5% for the latter (*P =* 0.577). • EFS and OS were not significantly associated with age
Bunin et al. (30)	6–20 y/o	29/43	Busulfan vs. total body irradiation containing conditioning regimens for children with acute lymphoblastic leukaemia	MAC TBI-based vs. MAC w/o TBI (BU-Cy vs. TB1-VP16)	• MSD • Haplo • MUD	BM, PBSC and UCB	EFS > 6y/o: BU 36.3%(11.2–62.7)/TBI 56.3%(27.2–77.6) *P* = 0.31
Burke et al. (45)	13-30 y/o	34/80	Comparison of survival in HSCT for ALL between children and AYA	Cyclophosphamide (120 mg/kg) with or without fludarabine (75 mg/m^2^) with total body irradiation (1,320 cGy): 96% busulfan/cyclophosphamide/fludarabine or etoposide/total body irradiation: 4%	71% MSD, 19% MUD, 10% MMUD	• 51% related UCB • 40% bone marrow • 10% PBSC	Reduction in OS for the AYA group hazard ratio [HR], 1.74, 95% CI, 1.04–2.95; *P =* 0.03 Cumulative incidence of TRM at 1 year was higher in the AYA patients compared with those age <13 years (28% [95% CI, 16–41%] vs. 14% [95% CI, 6–21%]; *P* 1/4.04)
Cahu et al. (46)	18–35 y/o	405/601	Impact of TBI-conditionning in adult T-ALL	523 patients (87%) received a high-dose TBI-based regimen 78 patients (13%) received chemotherapy-only regimens.	MSD or MUD	BM or PBSC	• 5-year LFS and OS was 41% (95% confidence interval (CI), 37–46%) and 45% (95% CI, 40–49%), respectively. • The overall 5-year NRM was 25% (95% CI, 22–29%) and the 5-year relapse incidence was 33% (95% CI, 29–37%). • Patients who received a TBI-based regimen had a 5-year LFS of 44% (95% CI, 40–48%) vs. 25% (95% CI, 15–35%) for chemotherapy-only regimen (*P =* 4 × 10–4). • In the TBI group, the cumulative incidence of grade II–IV acute GvHD at day 100 was 40% (95% CI, 35–45%) vs. 27% (95% CI, 16–38%) for the chemotherapy group (*P =* 0.02).
Dhédin et al. (27)	15–44 y/o	414/522	Role of allogeneic stem cell transplantation in adult patients with Ph-negative acute lymphoblastic leukaemia	• 10 patients received reduced-intensity conditioning regimen 17 patients were conditioned without total body irradiation • All other: MAC TBI based	• 139 MSD • 143 MUD • 92 10/10 • 38 9/10 • 13 UCB	BM in 184 patients, PBSC in 85, UCB in 13.	• Relapse Free Survival (HR, 0.80; 95% CI, 0.60–1.06; P 5.12) and OS (HR, 0.76; 95% CI, 0.57–1.02; P 5.069) were not significantly improved in the SCT cohort. • The lower cumulative incidence of relapse (HR, 0.50; 95% CI, 0.35–0.70; P 0.001) observed in the SCT cohort was counterbalanced by a higher Non-Leukaemia Relapse Mortality (HR, 1.46; 95% CI, 1.09 to 1.95; P 5.011) Advanced age and administration of a higher number of pretransplant consolidation cycles were both associated with a significantly higher post-transplant NRM
Friend et al. (34)	12–25 y/o	27/57	Impact of total body irradiation-based regimens on outcomes in children and young adults with acute lymphoblastic leukaemia	MAC TBI Based vs. MAC w/o TBI	MSD, MUD, MMUD, and Haplo	BM, PBSC	Patients that received a non-TBI-based regimen had lower 3-year EFS compared to those who received TBI: 52 vs. 77%, *P =* 0.03 Non-TBI-based regimens showed a higher 3-year cumulative incidence of relapse: 34 vs. 18%, *P =* 0.13
Hangai et al. (3)	10–29 y/o	1,386/1,993	Differences in clinical outcomes and complications across age groups of patients who underwent HSCT for ALL	MAC TBI-based, MAC w/o TBI, RIC	MSD, MUD, MMUD, and Haplo		5-year survival rates of children, adolescents, and young adults: 70% (95% confidence interval [CI], 66–74%), 64% (95% CI, 60–68%), and 64% (95% CI, 60–68%), respectively, TRM was significantly higher for adolescents and young adults compared with children (*P* < 0.001 and *P =* 0.005, respectively)
Kalaycio et al. (31)	Not specified	Unknown/115	BU- vs. TBI-based conditioning for adult patients with ALL	MAC TBI-based, MAC w/o TBI	MSD or MUD	BM or PBSC	EFS was better among patients transplanted with a TBI-based preparative regimen (*P =* 0.046) compared with regimens containing BU. No significant difference in the cumulative incidence of either acute or chronic GVHD in patients treated with TBI compared with those treated with BU (*P =* 0.56 and *P =* 0.63, respectively).
Kiehl et al. (47)	17–26 y/o	84/221	Outcome of ALL patients receiving a related or unrelated stem-cell graft from matched donors.	MAC TBI-based, MAC w/o TBI	MSD or MUD	BM or PBSC	TRM was similar in matched related and unrelated transplantation. Trend for higher incidence of severe grade acute GVHD in matched unrelated transplants in comparison with matched related transplants (*P =* 0.055). In the multivariate analysis, tendency toward improved DFS in patients 17–26 years of age
Marks et al. (29)	Not specified	Unknown/1521	Outcome of full-intensity and reduced-intensity conditioning for matched sibling or unrelated donor transplantation in adults with Philadelphia chromosome–negative acute lymphoblastic leukaemia in first and second complete remission		MSD, MUD, or MMUD	BM or PBSC	TRM for the older RIC group was comparable with that seen in the younger full-intensity cohort.
Michel et al. (39)	10–35 y/o	69/151	Single- vs. double-unit cord blood transplantation for children and young adults with acute leukaemia or myelodysplastic syndrome	MAC TBI-based, MAC w/o TBI	UCB 6/6 HLA-identical unit was preferred to a 5/6 HLA-identical and a 5/6 unit to a 4/6.	UCB	• Cumulative incidence of relapse was 14.9 ± 4.2% in the single-unit arm and 23.4 ± 4.9% in the double- unit arm (*P =* 0.21) • The overall incidences of acute and chronic GvHD did not differ, but chronic GVHD was more frequently extensive after double-unit UCBT. • The 2-year cumulative incidence of extensive chronic GVHD was 31.9 ± 5.7% after double-unit vs. 14.7 ± 4.3% after single-unit transplantation (*P =* 0.02).
Nagler et al. (48)	Not specified (>18 y/o)	Unknown/593	Comparison of haploidentical bone marrow vs. Matched unrelated donor peripheral blood stem cell transplantation with posttransplant cyclophosphamide in patients with acute leukaemia	MAC TBI-based, MAC w/o TBI, RIC	MUD or Haplo	BM for haplo and PBSC for MUD	• Risk of grade 2–4 acute GvHD (HR = 0.53, *P =* 0.01) and chronic GvHD (HR = 0.50, *P =* 0.02) was significantly lower in the haplo-BM group compared with the UD-PB group. • No significant difference between the study groups with respect to relapse incidence, non relapse mortality, leukaemia-fee survival, overall survival, or GvHD-free and relapse-free survival.
Peters et al. (35)	14–2 y/o	110/413	Total body irradiation or chemotherapy conditioning in childhood ALL	MAC TBI-based, MAC w/o TBI	MSD or MUD	BM, PBSC, UCB	OS was significantly higher following TBI vs. chemo conditioning, with a 2-year probability of OS of 0.91 (95% CI, 0.86–0.95) vs. 0.75 (95% CI, 0.67–0.81), respectively (*P =* 0.0001). Two-year EFS was significantly higher following TBI vs. chemo conditioning (0.86 [95% CI, 0.79–0.90] v 0.58 [95% CI, 0.50–0.66], respectively; *P =* 0.0001). Two-year CIR was 0.12 (95% CI, 0.08–0.17) following TBI and 0.33 (95% CI, 0.25–0.40) following chemo conditioning (*P =* 0.0001)
Peters et al. (49)	12–18 y/o	150/411	Comparing sibling donors with matched unrelated donors in childhood ALL	TBI-VP16	MSD, MUD, or MMUD	BM for MSD and BM or PBSC for MUD/MMUD	• No differences in incidence or severity of aGVHD were observed between MSD-HSCT and MUD-HSCT patients. • Extensive cGVHD occurred more frequently after MSD-HSCT. The incidence of grade 3–4 infection was significantly higher in the MUD group. The 4-year EFS rate after MUD-HSCT was similar to that after MSD-HSCT (0.67 ± 0.03 vs. 0.71 ± 0.05; *P =* 0.405)
Ruggeri et al. (41)	Not specified (>18 y/o)	Unknown/528	Comparison of outcomes after unrelated cord blood and unmanipulated haploidentical stem cell transplantation in adults with acute leukaemia	MAC TBI-based, MAC w/o TBI or RIC	Unmanipulated UCB or Haplo	UCB or unknown	Relapse was not statistically different between the two treatment groups: HR = 0.82, *P =* 0.31 NRM was not different between UCBT and Haplo recipients: HR = 1.23, *P =* 0.23. After Haplo and UCBT, the probability of LFS at 2 years was 28 and 34% (*P =* 0.49)
Simonin et al. (38)	Not specified (<18 y/o)	Unknown/2584	Peripheral blood stem cell compared with bone marrow in hematopoietic transplantation for paediatric acute lymphoblastic leukaemia	MAC TBI-based, MAC w/o TBI	MSD or MUD/MMUD	PBSC or BM	3-year probability of OS was significantly higher after BM vs. PB transplantation (67%; 95% confidence interval (CI): 66–68 vs. 62%; 95% CI: 60–64%; *P =* 0.0004). 3-year probability of LFS was significantly higher after BM transplantation (59%; 95% CI: 58–60%) than after PB transplantation (54%; 95% CI: 53–55%; *P =* 0.0007). NRM was significantly higher after PB transplantation (hazard ratio (HR) 1.38; 95% CI: 1.04–1.83; *P =* 0.02) CIR at 3 years was similar between the two groups: 29% (95% CI: 28–30%) and 26% (95% CI: 24–28%) after BM and PB transplantation, respectively (*P =* 0.29).
Tracey et al. (26)	11–18 y/o	312/765	Effect of 4 commonly used transplantation conditioning regimens on leukaemia relapse, transplantation-related mortality, and overall survival	MAC TBI based	MSD, MUD/MMUD	PBSC, BM or CB	Risk of relapse was similar among the 4 treatment groups. Risk of transplantation-related mortality differed by conditioning regimen: those who received TBI > 1,320 cGy + Cy + etoposide were at greater risk for transplantation-related mortality. Age >10 years (HR, 1.93; 95% CI, 1.45–2.56; *P* < 0.001) was associated with a higher risk of transplantation-related mortality. Overall mortality risk also differed by transplantation conditioning regimen: Recipients of TBI > 1,320 cGy who received Cy + etoposide had a higher mortality risk compared with those who received Cy alone. Mortality risk was higher in patients age >10 years (HR, 1.05; 95% CI, 1.22–1.85; *P* < 0.001)
Wood et al. (15)	15–40 y/o	1,218/2,668	Survival improvements in adolescents and young adults after myeloablative allogeneic transplantation for acute lymphoblastic leukaemia		MSD, MUD/MMUD	PBSC or BM	Older patients having lower 5-year OS and LFS rates than AYAs, who in turn had lower OS and LFS rates than children AYAs having higher 5-year TRM than children and adults having higher TRM than AYA Older age was shown to be associated with poorer survival (hazard ratio [HR], 2.04 for older adults and 1.57 for AYAs vs. children, *P* < 0.001
Xue et al. (50)	10–18 y/o	36/70	Role of allogeneic hematopoietic stem cell transplantation (allo-HSCT), particularly haploidentical (haplo)- HSCT, in paediatric patients with Philadelphia chromosomepositive (Ph+) acute lymphoblastic leukaemia (ALL)		MSD, MUD, or MMUD, Haplo	BM or UCB	OS, EFS, and CIR rates were significantly better in the transplant arm in the high-risk group Haplo-HSCT showed a significant survival advantage in the high-risk group only.

*MAC, Myeloablative conditioning; RIC, reduced intensity conditioning; TBI, total body irradiation; MSD, Matched sibling donor; MUD, matched unrelated donor; MMUD, mismatched unrelated donor; haplo, haploidentical donor; BM, Bone marrow; PBSC, peripheral blood stem cell; UCB, unit of cord blood; CIR, cumulative incidence of relapse; LFS, leukaemia free survival; EFS, event free survival; OS, overall survival; DFS, disease free survival; NRM, non-relapse mortality; TRM, treatment related mortality; AYA, adolescents and young adults; ALL, acute lymphoblastic leukaemia; SCT, stem cell transplantation*.

## HSCT-Associated Complications and Supportive Care

Early toxicities associated with HSCT in AYAs remain a major issue. According to several studies, TRM in CR1 ranges from 10 to 30%, mostly due to GvHD and infection (12, 14–16, 45, 51).

A retrospective cohort study performed by the Japan Society for Hematopoietic Stem Cell Transplantation (JSHSCT) in 1,993 patients in Japan found a greater risk of NRM in AYAs with ALL after allogeneic HSCT (19%; 95% CI, 16–22%) compared to children (11%; 95% CI, 8.8–14%); *p* < 0.001 with infectious complications (24 vs. 4.2%, respectively) being the most common cause of death in AYAs (3). Similarly, Burke et al. identified significantly lower 5-year OS in the AYA group (*n* = 57; HR 1.74; 95% CI, 1.04–2.95; *P* = 0.03) after myeloablative allogeneic HSCT for ALL vs. the children group (*n* = 79). The inferior outcome was due to a 2-fold increase in TRM after 1 year (HR 2.23; 95% CI, 1.01–4.90; *P* = 0.05) in AYA patients compared to <13 years old patients, with rates being particularly high when bone marrow was used as graft source, while no age-related difference in relapse rate or acute GvHD was noted (45).

In a retrospective analysis of the CIBMTR, the outcome of ALL in children (*n* = 981), AYAs (*n* = 1,218), and older adults (*n* = 469) after myeloablative conditioning and allogeneic HSCT over almost two decades in paediatric and adult transplant centres was compared. The researchers noted parallel survival improvements in AYAs and children over time, yet survival remained inversely correlated with age (HR of 2.04, 95% CI 1.75–2.39, for older adults and 1.57, 95% CI 1.40–1.77, for AYA compared with children; *p* < 0.001). Again, TRM following both MSD or MUD transplantations was higher in AYAs compared with in children (HR, 1.66; 95% CI, 1.42–1.95; *p* < 0.001). The cause of this age-dependent increase in TRM remains unclear; it was speculated that disease- and age-related biology may play a role. Time-dependent effects seen during an observation period of 27 years were attributed to improved supportive care leading to a lower rate of early TRM over time (15).

Based on these findings, the reduction of TRM and use of attentive supportive care appear critical for successful HSCT in AYAs. Optimised peri-transplantation care with diligent infectious disease work-up and monitoring as well as infectious prophylaxis appear mandatory, particularly in this vulnerable age group.

Special attention should be paid also to immunosuppressive treatment after HSCT in these patients to reduce toxicity and infectious complications. Data in adult patients undergoing MSD HSCT, for example, suggest that the combination of mycophenolate mofetil and cyclosporine A is less toxic but similarly effective to methotrexate and cyclosporine A (52).

Both the Berlin-Frankfurt-Münster (BFM) 2002 and International BFM 2007 HSCT studies in patients with ALL <21 years old demonstrated the safety of less-intensive GvHD prophylaxis with Cyclosporine-A alone for patients transplanted from an MSD with bone marrow as the stem cell source, whereas Cyclosporine-A plus short-duration methotrexate remains the gold standard for GvHD prophylaxis in the adult HSCT setting regardless of donor type (42, 49).

Obesity has been identified as a risk factor for an adverse outcome in AYAs treated for ALL, with inferior DFS observed when BMI ≥ 30 kg/m^2^ (HR, 1.97; 95% CI, 1.51–2.57; *p* < 0.001) (13). Both relapse rate and NRM appear to be higher in obese AYAs—a fact that suggests an influence of metabolic parameters and altered pharmacokinetics on outcome (13). Obesity in adults undergoing allogeneic HSCT for hematologic malignancies has not been found to be a deleterious effect on OS or relapse, while some studies have shown increased NRM in adult obese patients (13). Thus, the negative effect of obesity on OS and relapse might be specific to the AYA population. underlining again the specific needs of this group of patients.

Treatment intensity and cumulative toxicity burden before the HSCT procedure must be considered by clinicians as it impacts on overall results. Therefore, the development of novel treatment strategies with fewer toxic side effects than standard chemotherapy may improve outcomes for AYAs with ALL in the future.

## Late Effects After Oncological Treatment and Allogeneic HSCT in AYAs

Given that there are very few studies focusing on allogeneic HSCT in AYAs and intensive treatment is usually required to achieve remission, the late effects after HSCT in this age group remain partially obscure. A retrospective Childhood Cancer Survivor Study comparing 10,397 survivors with 3,034 siblings revealed a cumulative incidence of chronic health conditions of up to 73.4% in adults 30 years after their cancer diagnosis (53). Compared to other childhood cancer survivors, those who received an MSD or MUD bone marrow transplant for haematologic malignancies demonstrated a significantly elevated risk of poor general health (relative risk [RR], 3.2 and 2.0, respectively; *p* < 0.01), functional impairment (RR, 7.8 and 8.4, respectively; *p* < 0.01) and activity limitations (RR, 5.9 and 10.1, respectively; *p* < 0.01) (54). With improvements in survival over the years and increasing numbers of younger survivors, this disparity becomes progressively more relevant.

In a retrospective study published by Burke et al. comparing outcomes after HSCT in children vs. AYA B-ALL patients, no correlation between the risk of acute GvHD and age was noted (45). Nevertheless, a non-significant trend toward a higher RR of cGvHD in AYA vs. in children was found in a multivariate analysis (RR, 2.73; 95% CI, 0.93–7.96; *P* = 0.07) (45). Similarly, Hangai et al. compared outcomes after HSCT for ALL among children (age 1–9 years; *n* = 607), adolescents (age 10–19 years; *n* = 783), and young adults (age 20–29 years old, *n* = 603) retrospectively (3). They found significant age-dependent differences in the 1-year incidence rate of cGvHD following HSCT in children (24%; 95% CI, 21–27%) adolescents (28%; 95% CI, 24–31%) and young adults (32%; 95% CI, 29–36%; *P* < 0.001), but not in the rate of aGvHD (3).

The potentially heightened risk of cGvHD in AYAs is of utmost importance, as cGvHD is generally associated with considerable mortality and morbidity resulting in a significantly poorer quality of life and functional impairment. This poses a special challenge in AYA patients, who are in a critical phase of development, and raises physical, psychological, and social challenges that need particular attention. Vigorous screening for risk factors and regular function testing after allogeneic HSCT are required to detect such chronic health issues early (44, 55).

This pre-emptive approach also applies to other late sequelae observed in AYA patients treated with allogeneic HSCT, similarly to the approach for late toxicities seen in the oncology patient population; it involves equivalent follow-up and screening programmes as those suggested by the National Comprehensive Cancer Network. Studies on long-term outcome in paediatric patients and AYAs treated with chemotherapy and radiation have revealed an increased incidence of secondary malignancies of 4–17% 10 years after allogeneic HSCT for their primary malignancy (53, 56, 57). As these treatment modalities are part of the conditioning regimen, close monitoring for occurrence of secondary malignancies is mandatory during long-term follow-up.

Besides haematologic diseases such as myelodysplastic syndrome and secondary leukaemia (cumulative incidence of 3.8 at 6 years), solid tumours (cumulative risk of 11% after 15 years) are more prevalent after allogeneic HSCT, in children than in adults (57–59). Therefore, screening for cutaneous malignancies (cumulative incidence of 3.4–6.5% at 20 years) as well as lung, breast (HR of 10.8 10 years after HSCT, compared to United States Surveillance Epidemiology End Results (SEER) for expected rates) and thyroid cancer (RR of 4.8 in AYAs, compared to general population) is mandatory during long-term follow-up after HSCT. An even higher incidence of solid cancer is observed in patients who received TBI-based conditioning vs. those who received chemotherapy based-conditioning (56, 60, 61). The cumulative incidence of post-transplant lymphoproliferative disorder at 10 years after allogeneic HSCT is 1% and varies depending on risk factors such as a mismatched donor, T-cell depletion, GvHD and irradiation (62).

Endocrine effects are another special consideration after allogeneic HSCT in AYA, particularly when TBI is part of the conditioning regimen. An increased rate of thyroid and gonadal dysfunction with >90% risk of infertility, growth impairment and skeletal complications occur after chemotherapy and irradiation (63–65). Therefore, preventive measures and timely replacement therapy are important.

In addition, regular testing of pulmonary, renal, and cardiovascular parameters is advisable as these organs may exhibit chronic dysfunction after allogeneic HSCT detectable as abnormal pulmonary function test results, renal insufficiency, hypertension, abnormal electrocardiogram readings, and impaired cardiac function. Although these adverse effects are not exclusively observed in AYAs as a consequence of chemotherapy or irradiation therapy, early detection is crucial in order to mitigate disabilities (54).

Table 3 propose recommendations for follow-up examination after allegeneic HSCT in AYA patients in the context of ALL treatment [adapted from (55)].

**Table 3:** Recommended screening for AYA who underwent HSCT.

**Recommended screening/Prevention**	**6 month**	**1 year**	**Annually**	**Comments**
**Immunity**				Consider increased risk of cGvHD and infectious complications in AYA and adapt frequency and duration of regular monitoring
**Ocular**				
Ocular clinical symptom evaluation (particularly sicca symptoms)	1	1	1	
Ocular fundus exam	#	1	#	
**Oral complications**				Consider increased risk of SSC in cGvHD, avoid smoking, sugar and piercing
Clinical assessment	1	1	1	
Dental assessment	#	1	1	
**Respiratory**				
Clinical pulmonary assessment	1	1	1	
Smoking tobacco avoidance (active and passive)	1	1	1	
Pulmonary function testing/Chest radiography	#	#	#	
**Cardiac and vascular**				
Cardiovascular risk-factor assessment	#	1	1	
**Liver**				PCR monitoring in case of HBV and HCV infection
Liver function testing	1	1	#	
Serum ferritin testing		1	#	
**Kidney**				Avoid nephrotoxic medication
Blood pressure screening	1	1	1	
Urine protein screening	1	1	1	
Bun/creatinine testing	1	1	1	
**Muscle and connective tissue**				In cGvHD: joint motility testing, sclerotic changes of skin
Evaluation for muscle weakness	2	2	2	
Physical activity counselling	1	1	1	
**Skeletal**				Vitamin D and Ca++ substitution, physical exercise
Bone density testing (adult women, all allogeneic transplant recipients and patients at high risk for bone loss)		1	#	Risk factor: glucocorticoid treatment, calcineurin inhibitor exposure
**Nervous system**				
Neurologic clinical evaluation	#	1	1	
Evaluate for cognitive development and achievement of developmental milestones		1	1	
**Endocrine**				Consider hormonal replacement
Thyroid function testing		1	1	
Growth velocity and growth hormone function in children		1	1	
Gonadal function assessment (prepubertal men and women)	1	1	1	
Gonadal function assessment (postpubertal women)		1	#	
Gonadal function assessment (postpubertal men)		#	#	
**Mucocutaneous**				Adequate sunprotection of skin
Skin self-exam and sun exposure counselling	1	1	1	
Gynecologic exam in women	#	1	1	
**Second cancers**				Self exam; risk factor: UV exposure, TBI-based conditioning
Second cancer vigilance counselling		1	1	
Screening for second cancers		1	1	Include mammography in women after irradiation
**Psychosocial**				
Psychosocial/QOL clinical assessment	1	1	1	
Sexual function assessment	1	1	1	

*1, recommended for all patients; 2, recommended for patients with ongoing cGvHD/Immunosuppression; #, reassessment recommended for abnormal findings*.

## Social and Psychological Issues

A difficulty of treating AYA patients is that the cancer diagnosis and treatment occur in a critical stage of life. Treatment side effects such as weight changes, hair loss, and growth disturbances may be more difficult to cope with for patients in this age group than for younger or older patients. Adolescence and young adulthood is a period of developmental processes and psychosocial and hormonal challenges, where major aspects of life and future plans (developmental tasks of emerging adulthood) become more important (66).

The attainment of social, financial, and physical independence are three major aspects in the transition to adulthood—all of which can be affected by anti-cancer treatment including HSCT. This may hinder successful transition to adulthood and compromise individuals' long-term quality of life (67, 68).

AYAs with cancer are generally more dependent on family than are other AYAs, which makes it difficult to “cut the cord” from the parents (69, 70). They can have difficulties in continuing their education and occupation, with alterations needed to educational or career plans (71).

The feeling of social and medically imposed isolation due to absence from school and separation from friends can lead to loneliness, developmental discrepancy, and social disruption (67). The loss of friendships during therapy aggravates patients' reintegration into “normal life” (43).

This disruption of everyday life and patients' confrontation with their own mortality can bring fear, distress, and uncertainty (66, 71). Several studies have shown higher levels of depression, anxiety and distress in AYA patients with oncological disease compared with healthy peers (66, 72).

Non-compliance to treatments in this age group is a major problem (73). It is important to help AYAs to continue to live as normally as possible, have as much information as they need, and to be involved in treatment decisions and their own care, in order to support autonomy and to promote a trustful relationship (74, 75). Clinicians must consider that each patient is at a unique developmental point and, therefore, their needs differ (76).

During adolescence and young adulthood, intimate emotional, and sexual relationships are often formed, which can be complicated by a cancer diagnosis and treatment due to altered body image, social isolation, and the fear of rejection. Patients may consider themselves as unattractive and undesirable leading to decreased libido (77). Furthermore, the fear of infertility can have a negative influence on intimacy (75). Early management of sexual dysfunction can improve the situation and patients' quality of life.

Clinicians should consider all of the aspects above when treating AYAs, although there are no specific recommendations for those with ALL undergoing HSCT. In general, only a specialised, multidisciplinary team of health professionals (including specialised physicians, nurses, psychologists, and social workers) who can provide age-appropriate information and address the fears of this age group should treat AYA patients (78, 79).

Various evidence-based psychosocial interventions exist to reduce distress and help patients to cope with exceptional circumstances. Multidisciplinary programmes include peer-to-peer support to encourage relationships and skill-based and technology-based interventions tailored to the unique needs of AYAs facing cancer and HSCT (75, 79). Various studies emphasise that securing adequate social support is the most important coping strategy and resource for AYAs when facing cancer (74).

Moreover, to achieve the best outcomes, treatment should be administered in specialised centres with the highest expertise and the opportunity to enrol patients in clinical trials (73, 80).

## Fertility Issues

As described above, compromised reproductive function is a major and severe late complication in cancer survivors, which can lead to psychosocial distress and depression and has a significant influence on quality of life (1, 81–84).

Studies have shown that infertility caused by cytostatic drugs is dependent on the dosage and type of drug given and also on the patient's age at treatment (85–87).

Younger age (<10 years old) at the time of exposure to cytostatic drugs or radiotherapy is associated with a lower risk of premature ovarian insufficiency (POI) (87, 88).

The risk of infertility is high in patients receiving HSCT following a conditioning regimen of TBI, high-dose cyclophosphamide, melphalan, and busulfan (81, 82, 85). More than two thirds of patients who receive allogeneic HSCT develop gonadal dysfunction (85). In transplanted patients, the detrimental effects of HSCT and of previously received frontline chemotherapy play a synergistic role (87).

Although long-term recovery of gonadal dysfunction after HSCT has been reported, female patients who receive TBI have a high risk of a later-onset POI (87, 89). The ovarian damage is irreversible in most cases (90). Female patients who received TBI or high-dose cyclophosphamide prior to HSCT have a much higher miscarriage rate and increased risks of preterm delivery and delivery of low-birth-weight infants (84, 91, 92). Chemotherapy and/or TBI as conditioning typically lead to disruption of the hypothalamic-pituitary-gonadal axis and direct gonadal damage (77, 93).

There are a few studies showing a recovery of spermatogenesis after TBI prior to HSCT (86, 93). Nevertheless, TBI plays a central role in infertility in male as well as female patients.

Considering this high risk of infertility and the impact it might have on a patient's life, comprehensive age-appropriate counselling about the risks, options for fertility preservation and why certain interventions cannot be performed in some situations (for example, coelioscopy or transvaginal puncture in cases of severe neutropenia or thrombopenia) is absolutely essential to reduce distress and is recommended in several guidelines (81, 82, 93–95).

Numerous cancer survivors report a lack of information and the fear of infertility often leads to maladaptive coping strategies (94, 96). Therefore, ALL patients should receive proactive counselling even though their options for fertility preservation (especially for pre-pubertal or female patients) are quite limited, patients are frequently acutely ill at diagnosis, and the severity of the disease may not permit a delay of anti-cancer therapy.

Frontline therapy for ALL usually does not cause permanent gonadal dysfunction (93, 97). The crux of treating patients with ALL is that the indication for HSCT is not clear at diagnosis in most patients; therefore, patients have been treated already with several cytostatic drugs before fertility protective procedures can be performed. In post-pubertal boys, sperm cryopreservation is an effective method for fertility preservation, which can easily be organised in most cases (81). It should ideally be implemented before the start of cancer treatment. Emotional stress, azoospermia, or decreased sperm mobility are some of the reasons why sperm banking is unsuccessful (84). It is common that male patients with an oncological diagnosis have low sperm counts even before starting cytostatic treatment (93). For post-pubertal girls, fertility preservation options are very limited. The two options are embryo or oocyte cryopreservation for which hormonal hyperstimulation is necessary. As this causes a delay of cancer treatment of ~2 weeks, it is often not feasible in acutely ill patients. High oestrogen levels need to be avoided due to their potential side effects in cancer patients. In addition, invasive procedures are associated with a higher rate of complications, mostly due to infections and bleeding in pancytopenic patients (93). Ovarian tissue or ovarian cortex cryopreservation are still experimental in ALL or AML because low levels of leukaemic cells have been found in the ovarian tissue of mice and humans in studies, which might possibly increase the relapse risk after re-transplantation of the tissue (77, 93, 98, 99). Whether these methods will be available in the future after testing for MRD remains to be determined (93). In pre-pubertal patients, gonadal tissue conservation is the only recommended option. However, due to the high relapse risk after re-transplantation, there are currently no recommendations in clinical guidelines for this technique in patients with ALL (82).

Another difficulty in standardised, international recommendations for all patients undergoing HSCT for ALL is the significantly different access to fertility protecting procedures between countries and the financial coverage through different healthcare systems. Financial coverage should be mandatory for fertility protection in treatment-associated infertility. Many AYA patients with cancer describe a high financial burden. Affording fertility preservation procedures for future family planning often causes massive emotional distress (93). The costs of preservation, banking and of further processing when pregnancy is desired are often obstacles for cancer survivors (77).

## Immunotherapeutic Concepts to Reduce Toxicity and Improve Outcomes in AYA Patients

Wood et al. noted a higher rate of late relapse (>12 months from HSCT) in AYAs who received HSCT for ALL vs. the relapse rate in children (HR, 2.1, 95%CI 1.59–2.75; *p* < 0.001); this increased risk did not significantly change with time in long-term studies. This increased risk of late relapse may be associated with the unfavourable disease biology observed in AYA and adult patients and raises the question of how to optimise pre-transplantation therapy, immunosuppressive strategies and consolidation treatment in certain patient groups (15).

With the advent of targeted immunotherapies such as the bispecific anti-CD3/anti-CD19 T-cell engager blinatumomab, the toxin-conjugated anti-CD22 antibody inotuzumab ozogamicin, and CAR T-cell therapies, novel strategies have emerged to: (1) induce deeper remissions prior to HSCT; (2) substitute toxic chemotherapy elements in vulnerable patient groups; and (3) substitute the entire HSCT procedure by use of long-lasting CAR T-cell products. The companion papers in this supplement by Krauss et al. and Buechner with colleagues discuss these topics in detail for the paediatric ALL population. No studies with any of the abovementioned drugs have been performed solely in an AYA cohort; however, information regarding AYAs can be retrieved from combined paediatric/AYA studies and isolated adult studies.

### Blinatumomab

Blinatumomab is approved in both adults and children for several indications in B-cell precursor (BCP)-ALL patients. Common to both groups is the approval in patients with relapsed (second or higher relapse) or refractory (R/R) CD19^+^ Ph-negative BCP-ALL. Additionally, adults with Ph-positive ALL who have failed treatment with at least two TKIs and adults with Ph-negative ALL in CR1 or CR2 with persistent MRD >0.1% have an approved indication for blinatumomab. In children, blinatumomab has additional approval for first high-risk relapse as part of consolidation therapy. It is currently being investigated by several collaborative study groups as part of paediatric ALL frontline protocols.

In a systematic meta-analysis including 628 R/R ALL patients from six clinical trials, response rates to blinatumomab did not significantly differ between paediatric and adult patients (100). The multinational, randomised, Phase 3 TOWER study (ClinicalTrials.gov identifier: NCT02013167) examined the outcomes of 405 adults with Ph-negative R/R BCP-ALL (age range 18–80 years) randomised to either standard-of-care chemotherapy or blinatumomab (24). Blinatumomab induced deep remission in responding patients, which was associated with a significantly longer OS (median OS 7.7 months, 95% CI 5.6–9.6 in blinatumomab group vs. 4.0 months,95% CI 2.9–5.3 in the chemotherapy group; HR for death, 0.71; 95% CI 0.55–0.93; *P* = 0.01). The trial was prematurely stopped due to a significant OS benefit in the blinatumomab arm regardless of subsequent HSCT. In the pivotal Phase 2 ALCANTARA study (NCT02000427) of adult patients (*n* = 45, range 23–78 years) with Ph-positive BCP-ALL who were intolerant to TKIs, response to blinatumomab was observed in 36% of patients, with 86% of the responders being MRD negative (101). In the Phase 2 BLAST study (NCT01207388, *N* = 116 patients range, 18–76 years), 78% of adults with BCP-ALL in CR but with persistent MRD (≥10^−3^) achieved complete MRD response within one blinatumomab treatment cycle (102).

Very similar results were obtained in children age <18 years with R/R BCP-ALL in a pivotal Phase II trial (NCT01471782) (103) and the blinatumomab expanded-access RIALTO trial (ClinicalTrials.gov: NCT02187354) (104), including in toxicity-prone patients with Down syndrome (104). In two recent studies in children with a first high-risk relapse of BCP-ALL, the superiority of blinatumomab compared to standard chemotherapy was impressively demonstrated; both trials stopped prematurely due to significantly better outcomes in the blinatumomab arm, including the fraction of patients eligible for subsequent HSCT and a lower rate of severe toxicity (105, 106).

Taking these findings together, blinatumomab induces MRD-negative responses in a substantial fraction of patients with MRD persistence or R/R disease, both in children and adults. Although distinct toxicities related to blinatumomab occur (immune-effector-cell–associated neurotoxicity syndrome and cytokine-release syndrome), blinatumomab is generally (and compared to alternative intensive chemotherapy) well-tolerated, with fewer serious adverse events compared to intensified chemotherapy. There are no data indicating that the AYA group would respond differently. Blinatumomab may be an alternative or supplement to current chemotherapy approaches to reduce toxicity before HSCT in high-risk AYA patients.

### Inotuzumab Ozogamicin

In the Phase 3 INO-VATE trial (NCT01564784), adult patients (age range 18–78 years) with R/R CD22^+^ BCP-ALL were randomised to either receive inotuzumab ozogamicin or standard-of-care chemotherapy (23). Patients treated in the inotuzumab ozogamicin arm had a significantly higher response rate (CR, 80.7 vs. 29.4%, respectively; *p* < 0.001), higher rate of MRD-negative remission (78.4 vs. 28.1%, respectively; *p* < 0.001) and were more likely to proceed to HSCT directly after their treatment course (48 vs. 32%, respectively; *P* = 0.12) than were patients in the chemotherapy arm. Non-haematological toxicities were mainly related to the liver, with veno-occlusive disease occurring in 15% of patients; the vast majority (90%) in patients proceeding to HSCT.

Inotuzumab ozogamicin has been used sporadically in children with R/R BCP-ALL in compassionate-use programs, as recently published (107, 108). CR was achieved in 67% of patients, with the majority (71%) of responders being MRD negative. Of note, responses were observed irrespective of cytogenetic subtype or number or type of prior treatment regimens. However, 52% of the patients who proceeded to HSCT post inotuzumab ozogamicin developed veno-occlusive disease. The efficacy and safety of inotuzumab ozogamicin as monotherapy or in combination with chemotherapy is currently being systematically investigated in children in a Phase I/II study (ITCC-059; EudraCT Number: 2016-000227-71) (109).

Similarly to the pattern observed with blinatumomab, the efficacy and toxicity profile of inotuzumab ozogamicin in adults and children are similar and do not indicate that AYAs would have distinct outcomes or profiles. However, veno-occlusive disease is one of the major complications seen after inotuzumab ozogamicin use and needs to be monitored closely and considered early in AYA patients, especially in the context of TBI-containing conditioning as used in ALL.

### Chimeric Antigen Receptor T-Cell Therapy

Most published studies investigating CAR T-cell therapy in BCP-ALL included patients up to the age of 25 years, or older patients. The companion paper by Buechner and colleagues in this supplement discusses CAR T-cell trials in detail. The pivotal ELIANA Phase II trial (NCT02435849) (25), which led to the approval by the Food and Drug Administration and European Medicines Agency of tisagenlecleucel as the currently only CD19 CAR T-cell therapy for R/R BCP-ALL, enrolled 75 patients in the age range 3–23 years (25). Remission rate by month 3 after infusion did not differ across age groups and was 81% (overall remission rate) for the entire cohort. In a combined analysis of two similar tisagenlecleucel trials (ENSIGN [NCT02228096] and ELIANA) focusing specifically on AYA patients aged 18–25 years old (*n* = 20), rates of adverse events were comparable to younger age cohorts and did not indicate that AYA patients are at higher risk of adverse events after a single infusion of tisagenlecleucel (110). A recent meta-analysis on CAR T-cell therapy in ALL did not find significant differences in outcomes when paediatric and adult data were compared (111). Indeed, the current approval of tisagenlecleucel includes AYA ≼ 25 years of age with either a second or higher relapse, a relapse post HSCT or who have a refractory BCP-ALL.

Beside its efficacy in inducing remissions across all age groups, all cytogenetic risk groups and in patients with previous HSCT, tisagenlecleucel may also have the potential to induce sustained remissions without consolidative HSCT because it can persist for months and years in patients and thereby provide disease control, at least against CD19^+^ disease. Toxicities in AYAs are well-documented (110). Still, long-term data, both on outcomes and toxicities, are sparse and further focus is needed on the AYA group. Moreover, antigen loss or lineage switch (in patients with *KMT2A*-rearrangements) are currently intrinsic limitations of targeted immunotherapies (see the review by Buechner et al. in this Frontiers supplement).

## Discussion and Conclusion

The data reviewed here reveal the special and dedicated attention that AYA patients require. Precisely defining the indication for allogeneic HSCT in this age group is fundamental, particularly because ALL in AYA patients is often associated with high-risk genetic abnormalities and refractory disease. Therefore, currently the percentage of AYA patients requiring HSCT in CR1 remains higher than that in paediatric patients <15 years old.

Careful monitoring and management of early toxicities associated with intensive chemotherapy and subsequent allogeneic HSCT is fundamental. AYA patients appear especially prone to developing fatal infectious complications. Reduced compliance with infectious prophylaxis regimens and neglect in reporting the clinical signs of infections in this age group may contribute to the dismal outcome and should be considered and addressed with the patient while on immunosuppressive treatment and during regular follow-up visits. In addition, late effects on functional impairment also remain major issues in this cohort. In fact, given the higher incidence of secondary malignancies and organ dysfunctions seen after irradiation, careful and systematic follow-up of these patients should be provided (Table 3).

For most patients with malignant haematological diseases, there is neither an optimal timing nor method for fertility preservation. All patients should be proactively counselled about their infertility risk and possible fertility protective options both before and after HSCT. A referral to reproductive specialists after HSCT is strongly recommended, especially if no fertility protection was performed before the start of therapy. All interventions must be provided with careful psychological support to try to avoid depressive crisis and feelings of loneliness with a dramatic loss of social links.

The development of less-toxic transplantation modalities associated with novel treatment strategies before HSCT associated with fewer adverse effects should be thoroughly investigated in the future. Indeed, the severe adverse events that characterise the treatment of high-risk ALL in this fragile cohort of patients might be mitigated by the introduction of new immunotherapies. Both inotuzumab ozogamicin and blinatumomab are promising drugs to induce MRD negativity in patients with chemo-refractory BCP-ALL clones and may help to put AYA patients into “transplantable” deep remissions with less toxicity than conventional chemotherapy.

Given the severity of acute and chronic adverse events and long-term physical and psychological sequelae detected in AYA patients, dedicated prospective, and comparative studies are urgently needed. The increasing accessibility of new immunotherapeutic approaches allows evaluation of their significance in the treatment of AYA patients. Of particular interest will be the question of whether these agents are able to induce long and stable CRs without HSCT, as is currently being investigated in the CASSIOPEIA trial (NCT03876769) of CAR T-cell therapy for patients aged 1–25 years with high-risk *de novo* BCP-ALL, with the goal to see whether HSCT can be substituted.

In conclusion, in light of their unique needs, we strongly recommend that AYA patients receive treatment in dedicated centres with multidisciplinary expert teams. Such multidisciplinary approaches require different specialised physicians working beside one another, including the haematologist, stem transplantation expert, psychologists, physiotherapists and social workers familiar with the requirements of AYAs as outlined in Table 4 [adapted from (47)].

**Table 4:** Multidisciplinary approach in treatment of children and AYA undergoing HSCT.

**Pretransplant workup**	**Paediatric haemato-oncologist**
	**Paediatric/adult HSCT physician**
	**Fertility/reproductive counselling**
	**Psychologist specialised in paediatric/adolescent care**
	**Irradiation department experienced in treatment of children and adolescents**
	**Diagnostic and reference laboratories (including MRD and chimerism assessment)**
**Medical care during HSCT**	**Enrolment of pts in clinical studies**
	**Paediatric haemato-oncologist**
	**Paediatric/adult HSCT physician**
	**Irradiation department experienced in treatment of children and adolescents**
	**(paediatric) infectious disease (ID) specialists**
	**Counselling of physicians of otder (paediatric) subspecialties**
	**Specialised paediatric/adolescent intensive care unit**
	**Radiology department**
	**Pharmacologists**
	**Nurses specialised in paediatric/adolescent care**
	**Psychologist specialised in paediatric/adolescent care**
	**Physiotderapist**
**Social support**	**Family/parents/siblings**
	**Continued (virtual) contact witd friends/peers**
	**Teachers in clinic and possibility of online teaching**
	**Art/music tderapists**
	**Social workers**
	**Spiritual assistance**
**Rehabilitation and transition after HSCT**	**Paediatric/adult HSCT physician**
	**Physical and occupational tderapist**
	**Psychologist specialised in paediatric/adolescent care**
	**Social workers**
	**Teacher**
	**Career/life counsellors**
**Follow-up after HSCT**	**Paediatric/adult HSCT physician**
	**Close ID and GvHD monitoring program**
	**Meticulous ID prophylaxis (encapsulated organisms, PJP, CMV)**
	**Vaccination program post HSCT**
	**Specialised follow up care and late effects clinic (paediatric and adult coop) including consultants:**
	**e.g., neurologist, pulmonologist, ophtdalmologist, dentist, cardiologist, nephrologist, gastroenterologist**
	**Endocrinologist including bone densitometry and fertility specialists**
	**Screening program for secondary malignancies**
	**Transition in long-term and adult survivorship clinics**
	**Psychosocial support witd neuropsychological evaluation and QOL assessment**
	**Physical tderapist and long-term training programs**
	**Career/life counsellors**

*ID, infectious diseases; CMV, cytomegalovirus; PJP, Pneumocystis jirovecii pneumonia; QOL, quality of life*.

## Author Contributions

All authors listed have made a substantial, direct, and intellectual contribution to the work and approved it for publication.

## Funding

This study received funding from the St. Anna Children's Cancer Research Institute, Vienna, Austria. The funders were not involved in the study design, collection, analysis, interpretation of data, the writing of this article, or the decision to submit it for publication.

## Conflict of Interest

JB has participated in advisory boards organised by Novartis, Janssen and Gilead, and has received consulting/speaker's bureau fees from Novartis. The remaining authors declare that the research was conducted in the absence of any commercial or financial relationships that could be construed as a potential conflict of interest. The handling editor declared a shared consortium with the authors at time of review.

## Publisher's Note

All claims expressed in this article are solely those of the authors and do not necessarily represent those of their affiliated organizations, or those of the publisher, the editors and the reviewers. Any product that may be evaluated in this article, or claim that may be made by its manufacturer, is not guaranteed or endorsed by the publisher.
